# Multidetector computed tomography has replaced conventional intravenous excretory urography in imaging of the kidneys: A scoping review of multidetector computed tomography findings in renal tuberculosis

**DOI:** 10.4102/sajr.v22i1.1283

**Published:** 2018-02-16

**Authors:** Ntombizakhona B.A. Mthalane, Nondumiso N.M. Dlamini

**Affiliations:** 1 Department of Radiology, College of Health Sciences, Nelson R. Mandela School of Medicine, University of KwaZulu-Natal, South Africa

## Abstract

**Background:**

Tuberculosis (TB) is a worldwide infectious disease burden, especially in non-developed countries, with increased morbidity and mortality among human immunodeficiency virus (HIV)-infected patients. Extrapulmonary TB is rare and renal TB is one of the commonest manifestations. The end result of renal TB is end-stage renal disease; however, this can be avoided if the diagnosis is made early. The diagnosis of renal TB is challenging because of the non-specific presentation and low sensitivity of clinical tests. Although the sequel of TB infection in the kidney causes varying manifestations depending on the stage of the disease, multidetector computed tomography (MDCT) is capable of demonstrating early findings. We performed a 20-year scoping review of MDCT findings in renal TB to promote awareness.

**Aim:**

To identify specific MDCT imaging characteristics of renal TB, promote early diagnosis and increase awareness of the typical imaging features.

**Methods and material:**

We searched published and unpublished literature from 1997 to 2017 using a combination of search terms on electronic databases. We followed the Joanna Briggs Institute guidelines.

**Results:**

A total of 150 articles were identified, of which 145 were found through electronic search engines and 5 were obtained from grey literature. Seventy-nine articles that fulfilled our inclusion criteria were reviewed. These included original research, case reports, literature review, organisational reports and grey literature.

**Conclusion:**

Multidetector computed tomography can reproduce images comparable with intravenous excretory urography; together with advantages of being able to better assess the renal parenchyma and surrounding spaces, it is important in suggesting the diagnosis of renal TB and clinicians should consider including MDCT when investigating patients with recurrent urinary tract infection not responding to usual antimicrobial therapy.

## Background

Tuberculosis (TB) is the commonest communicable infectious disease worldwide and continues to be a serious burden in non-developed countries, being among the top 10 causes of death worldwide.^1,2^ South Africa is one of the top 6 countries in the world with the highest incidence of TB.^1^ Infection with human immunodeficiency virus (HIV) and acquired immunodeficiency syndrome (AIDS) increases the risk of contracting TB infection and also increases mortality from the disease.^1,3,4^ HIV-infected patients contributed to 55% of notified TB cases in 2015.^1^ The incidence of multidrug-resistant and extremely drug-resistant TB is seen to be on a rise in South Africa according to the World health Organization (WHO). The use of a more sensitive diagnostic test, that is, GeneXpert, allowing early diagnosis and initiation of treatment has been implemented in the call to stop the TB epidemic. One of the main goals of the WHO is to end the global TB epidemic and one of the implementations includes preventative measures such as the use of prophylactic treatment for children and HIV-infected patients.^1^

Tuberculosis is usually caused by infection with *Mycobacterium tuberculosis*; however, there are other described mycobacteria.^4,5,6,7,8,9,10,11^ TB can affect any system in the body and pulmonary TB is the most common form.^2,5,12,13^ Extrapulmonary TB is uncommon; however, co-infection with HIV or other immunocompromised states increases susceptibility.^5,14^ Urogenital TB is one of the common forms of extrapulmonary TB, with the kidney being the commonest site of infection, accounting for 15% – 20% of cases of extrapulmonary TB.^5,10,13,15,16^ Renal TB may occur as part of disseminated infection or as localised disease.^4,8,21^ The spread of infection to the kidney is almost always haematogenous from a pulmonary source with inoculation of mycobacteria in the renal cortex forming granulomas.^7,16–21^ These are invisible on imaging and, in the immunocompetent state, may remain indolent for 5–25 years; however, reactivation may occur during the immunocompromised state, resulting in enlargement and coalescence of the granulomas which then rupture into the renal tubular system.^7,16,22–26^ Some literature report a latent period of up to 40 years.^10,11,16^ Renal TB is particularly rare in children because of the long latent period and it is even rarer in children below 5 years of age.^8,9,11,18,23,27^ Renal TB has been reported to be common in young adults with a male predominance.^5,9,11,17,27^

The diagnosis of renal TB is difficult and is often delayed because the presence of constitutional symptoms is uncommon, clinical symptoms are non-specific and clinical tests have a low sensitivity.^11,13,15,16^ Patients may present with haematuria, flank pain and sterile pyuria.^5,8,11,16,27^ Renal TB should be strongly suspected in patients with sterile pyuria and recurrent urinary tract infection not responding to normal regimens of antimicrobial treatment, especially in endemic areas.^14,15,21,24,26^ Delayed diagnosis of renal TB often results in end-stage renal disease (ESRD) either because of parenchymal destruction or as a complication of chronic obstruction. This increases morbidity and mortality from the disease.^14,15,24,28^ Although radiological manifestations of renal TB are variable,^9,12,15,27^ multidetector computed tomography (MDCT) may aid in early diagnosis of the disease and early initiation of treatment and thus prevention of irreversible complications.^2,11,22,25,29^

A spectrum of renal pathology ranging from papillitis which eventually causes papillary necrosis, cavitation and spread of infection into the pelvi-calyceal system may occur. Extension of infection into the renal pelvis may result in pyonephrosis. Urothelial infection and inflammation subsequently progress to fibrosis and the formation of strictures. The ureter often has multiple strictures, which is pathognomonic and strictures in the distal ureter may result in megaureter.^2,6,11,16,29^ Distal ureteric strictures may be secondary to vesicoureteric reflux. Strictures at the infundibulum, renal pelvis and pelvi-ureteric junction are also common. Varying patterns of hydronephrosis and hydrocalicosis may occur depending on the site of obstruction.^2,7,10,11,12,29^ Urinary bladder involvement causes interstitial cystitis and granulomas may form in the bladder wall mimicking tumour; the end result is that of fibrosis of the bladder wall and reduced bladder capacity.^6,7,13,16,26^ The end result of chronic infection of the kidney is renal tissue destruction, which is subsequently replaced by dystrophic calcifications and autonephrectomy, also known as ‘putty kidney’.^4,12,16,26,30^ Calcifications in the wall of the ureter and in the bladder wall may also be seen.^6,16,17,30^ Renal TB infection may also spread beyond the renal capsule and form masses, also known as ‘pseudotumour’, which can be confused with renal malignancy.^2,7,12,17,27,29^ Rarely, renal TB may present with single or multiple parenchymal nodules without involvement of the renal collecting system, known as the pseudotumour type.^7,12,29^ Perinephric abscesses which may further complicate with formation of renal sinuses and fistulae are very rare.^6,7,11,26,29^ The presence of findings related to disseminated TB may be seen through computed tomography (CT) imaging, which may support the diagnosis of renal TB, that is, necrotic or calcified lymph nodes, hepatic and splenic granulomas, adrenal calcifications as well as spinal abnormalities.^2,7,10^ Patients with pulmonary TB and disseminated TB infection may rarely present with renal failure without any evidence of disease in the kidneys, as a result of interstitial nephritis.^4,7,11,20^

Because of haematogenous seeding of mycobacteria to the kidneys, both kidneys are usually affected although clinical and radiological assessment of the kidneys may show pathology in one kidney. It is therefore important to carefully examine the opposite side for subtle changes.^16,21,26^ Patients with renal TB require close monitoring after initiation and completion of treatment because healing occurs by fibrosis resulting in progression of ureteral and pelvic strictures.^2,15,24,26^ Six-monthly follow-up with renal ultrasound (US) for a period of up to 2 years is recommended.^15^ Disease process and spread is almost always in a descending pattern; however, cases of ascending spread of infection from primary genital TB and also rarely as a complication of Bacillus Calmette–Guérin (BCG) injection into the bladder to treat early stage bladder cancer have been reported.^2,8,11,12,16^ Most patients with renal TB have a normal chest X-ray or may have scarring from previous infection.^10,11^

The diagnosis of renal TB poses a challenge because of the non-specific presentation as well as the intermittent and low yield of the mycobacteria in the urine resulting in a low sensitivity of urine tests. Three early-morning urine specimens for Acid- Fast Bacilli (AFB) culture and histology is the gold standard for the diagnosis;^8,13,16,19^ however, urine culture demonstrates a low sensitivity of 30% – 40%.^15^ Some authors suggest more specimen collection, up to 5 and even up to 10 specimens.^13,23^ Polymerase chain reaction (PCR) is reported to be more sensitive.^8,11,20,29,31^ The diagnosis of renal TB requires a high index of suspicion and radiologists play a critical role.^6,7,10^

Computed tomography is readily available, and in the modern era, computed tomography urography (CTU) surpasses traditional intravenous excretory urography (IVU) in renal imaging because it allows comprehensive assessment of the renal parenchyma and surrounding spaces and structures, and better assessment of the urothelial and urinary bladder wall.^3,6,22,32^ An additional advantage of CT is the assessment of renal function.^29,33^ Intravenous excretory urography, however, has a higher sensitivity in the assessment of early irregularity of the calices (moth-eaten calices) and early changes in the mucosa of the urothelium (mucosal ulcerations),^10,17,24,27^ but does not allow accurate assessment of the renal parenchyma and surrounding spaces and structures.^6,11^ Other advantages of MDCT include the use of reformatted images such as multiplanar reconstruction (MPR), which includes curved MPR to straighten or visualise the entire length of the ureter on the coronal view; the use of maximum intensity projection (MIP) and three-dimensional (3D) reconstruction.^6,22,32^ In addition, using a bone display window when assessing the urinary tract on excretory phase imaging eliminates glare from high-density structures and thus helps with the assessment of calcifications and small urothelial lesions.^6^ All these technical advances can help optimise the assessment of the renal tract and its complex anatomy as well as help enhance pathology on MDCT.^6^ High radiation dose to the patient is the major concern in multiphase CT imaging of the urinary tract.^22,26,29,32,34^

Awareness of the MDCT features of renal TB is important as these may be subtle and unless one actively searches for these features, they may be easily missed.^2,3,7^ There are several case reports and literature reviews pertaining to MDCT imaging findings in renal TB. Some original research has been conducted across the world; however, according to our knowledge, there is no research identified in Africa regarding the subject. We performed a scoping review of the MDCT findings of renal TB, covering a 20-year period, to map these findings and emphasise the importance of meticulously searching for them as well as to promote awareness, especially in patients with known pulmonary TB or disseminated TB infection. We also reviewed the CT imaging protocols that are most appropriate for the diagnosis while considering the effects of high radiation dose acquired from MDCT. Use of appropriate MDCT renal protocol will help improve the sensitivity of MDCT in diagnosing renal TB.

## Review question

Multidetector CT is the modern imaging modality of choice in the diagnosis of renal TB, but unless one meticulously searches for specific features especially in early disease, these can be easily missed, thus subjecting the patient to the detrimental result of end-stage renal failure. Early MDCT findings of renal TB would include papillary necrosis, cortical granulomas, renal cavitation and abscesses, thickening and enhancement of the urothelium associated with strictures of the infundibulum, renal pelvis and ureter, irregularity of the urothelium involving the renal calices and ureter, non-uniform hydrocalicosis as well as hydronephrosis. It is unclear if radiologists interpreting MDCT images of patients with or suspected with TB infection would actively look for renal involvement.

## Objectives

The objectives of the study are as follows:

- to systematically scope the literature on MDCT imaging of renal TB
- to identify specific MDCT imaging characteristics of the disease process, promote early diagnosis and increase awareness of the typical imaging features
- to generate a comprehensive and well-defined list of the MDCT imaging findings using a chart, which will help guide radiologists in making a more accurate diagnosis
- to review the current MDCT imaging protocol for suspected renal TB that will increase the accuracy of CT in making the diagnosis.

## Methods

A scoping review is a newer and sound method for mapping areas of research and presenting results in an accessible format for knowledge users. Although it does not entail quality assessment, it is a rigorous and systematic approach to knowledge synthesis. It provides an overview of the existing literature and can help in identifying the fields in which more research might be necessary in the future.

We conducted a scoping review to examine the literature on MDCT findings of renal TB. This synthesis method will allow us to explore the broad topic of knowledge from different studies and literature. This scoping review proposal protocol was approved by the University of KwaZulu-Natal Postgraduate Office. The study did not require ethical approval.

## Steps of scoping review

Two reviewers independently collected literature published from January 1997 to June 2017, pertaining to the aims and objectives of the study. We followed the scoping review methods outlined by the Joanna Briggs Institute, which proposes the following framework for the structure of scoping review: (1) Title, (2) Background, (3) Review question/objective, (4) Inclusion criteria, (5) Search strategy, (6) Results, (7) Discussion and (8) Conclusion including implication for research practice.^35^

The steps followed in performing the scoping review include the identification of a research question, searching for relevant literature, categorising the topics of included literature, charting and analysis of the selected literature and consulting experts in the research fields, including librarians.

### Identification of relevant literature

A comprehensive search to identify both published and unpublished (grey) literature was performed. Identification of studies and literature relevant to this scoping review was achieved by searching electronic databases of the published and unpublished literature, which included MEDLINE, PubMed, ResearchGate, Cumulative abstracts and case reports, EBSCOhost and Google Scholar.

Our search strategy involved using a combination of search terms that were determined with input from the two reviewers. These included renal TB imaging, MDCT imaging of renal TB, MDCT + renal TB, extrapulmonary TB imaging and granulomatous nephritis. Input from University of KwaZulu-Natal research librarians was used.

### Selection of literature

The selection of data included literature review articles, original research, case reports and health organisation reviews and reports backdated from January 1997 to June 2017. Review articles, case reports and original research pertaining to renal TB and radiological and non-radiological publications were included. Current reports on the TB pandemic in the world and in South Africa also fulfilled our inclusion criteria. The study population included male and female patients of all age groups and there was no geographic limitation. We only selected articles that were published in English. Literature focusing on other forms of TB involving other parts of the body, excluding renal TB in the discussion, did not fulfil our inclusion criteria. Literature outside our study period was also excluded.

### Data extraction and charting of data

Windows Excel was used to capture and analyse data as per our inclusion and exclusion criteria. The spread sheet was designed to fulfil the aims and objectives of our study. A flow chart was used to summarise the data analysis.

## Results

**FIGURE 1:**
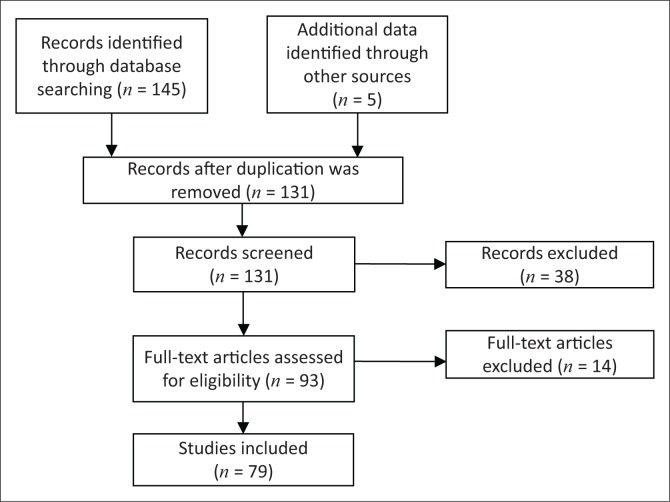
Flow chart showing the selection of articles reviewed.

The systematic process of selection of the articles that we reviewed is demonstrated in Figure 1. A total of 150 articles were identified, of which 52 articles were excluded because they did not fulfil the inclusion criteria. Reasons for exclusion were the following: articles published before 1997, articles that discussed renal TB outside the MDCT context (i.e. ultrasound, plain X-ray, magnetic resonance imaging and nuclear imaging), articles discussing extrapulmonary TB but renal TB was not part of the discussion and articles published in languages other than English. All included studies were reviewed and charted independently by two reviewers.

## Discussion

**FIGURE 2:**
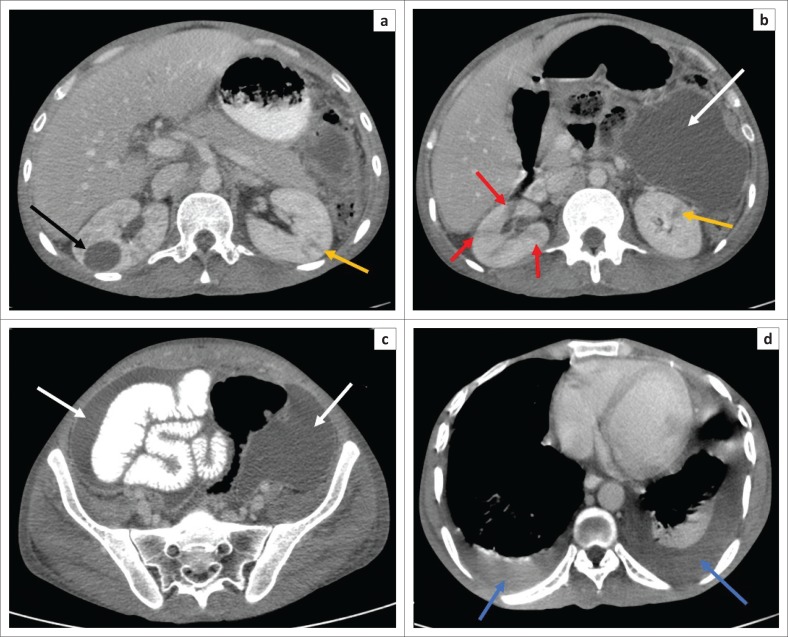
Post-contrast scan (venous phase) computed tomography (CT) abdomen: (a–d) Several small wedge-shaped areas of hypoattenuation in the right kidney in keeping with pyelonephritis (red arrow). Few similar areas are noted in the left kidney (yellow arrow). Large fluid-filled cavity in the right renal mid pole (black arrow). Moderate ascites with associated thickening and enhancement of the parietal peritoneum (white arrows) in keeping with peritonitis. There is also bilateral pleural effusion with associated relaxation atelectasis in the posterior left lower lobe (blue arrows).

**FIGURE 3:**
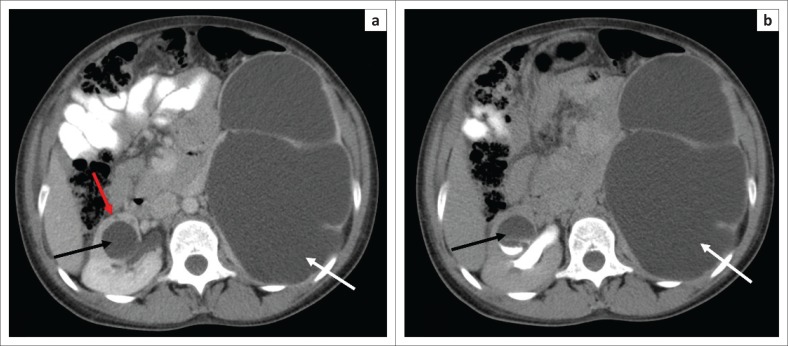
(a) Venous phase computed tomography (CT) abdomen demonstrates severe left hydronephrosis (white arrow) because of stricture in the pelvi-ureteric junction, with complete effacement of the renal parenchyma. A parenchymal cavity (black arrow) is present in the right kidney with surrounding heterogeneous low attenuation (red arrow) suggestive of pyelonephritis. (b) Delayed phase CT abdomen demonstrates contrast filling of the right renal cavity suggestive of communication with the calyceal system. The left kidney is non-functional.

**FIGURE 4:**
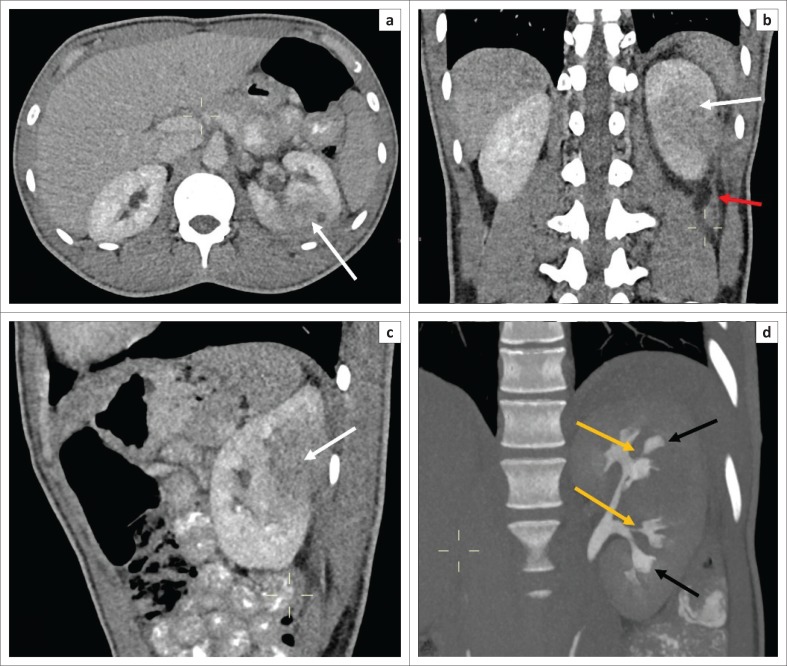
Post-contrast computed tomography (CT) abdomen, venous phase (a – axial, b – coronal, c – sagittal) and d – delay renal phase (MPR coronal) – a, b and c demonstrate a large heterogeneously enhancing left renal mass with a small cystic area (white arrow). (b) There is invasion of the Gerota’s fascia with associated thickening and enhancement (red arrow). (d) There is stenosis of the infundibula (yellow arrows) with slight focal caliectasis and distortion of the calices (black arrows).

**FIGURE 5:**
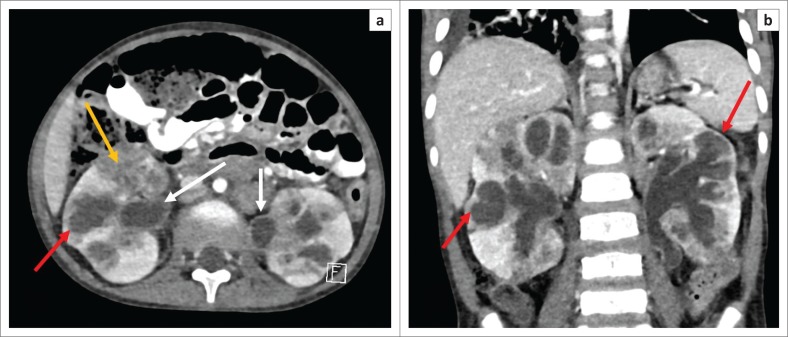
Post-contrast computed tomography (CT) (axial, coronal) shows: (a and b) bilateral hydronephrosis with non-uniform dilatation of the calices with areas of cortical thinning (red arrows). The calices are irregular and there is diffuse urothelial enhancement (white arrow). There are also patchy heterogeneous areas of reduced enhancement of the renal parenchyma with mass like areas of confluence (yellow arrow). The kidneys are enlarged.

The early MDCT findings of renal TB include papillary necrosis which is seen as small poorly marginated areas of hypoattenuation on the nephrographic phase at the tip of the medullary pyramid which gradually extends to involve the entire renal medulla (Figure 2). Detachment of the necrotic papilla may produce the appearance of a ‘signet ring sign’, seen on the excretory phase as a filling defect in the renal calyx with contrast seen tracking at the periphery of the pyramid.^6^ Wedge-shaped areas of low attenuation or hypoperfusion involving the renal cortex and medulla which cannot be differentiated from other causes of acute pyelonephritis may be seen. This is because of localised inflammation with vasoconstriction and oedema (Figure 2).^29,36^ Cavities may form in the renal parenchyma appearing as low-attenuating fluid-filled non-enhancing structures on all phases that fill with contrast on the excretory phase because of their communication with the renal collecting system (Figure 3). These may rupture into the perinephric space, forming perinephric abscesses which are seen as rim enhancing hypodense areas with associated thickening of the Gerota’s fascia.^6,29^ Small cortical granulomas may be seen on the corticomedullary and nephrographic phases as small rounded minimally enhancing hypoattenuating lesions in the renal cortex.^3,6,33,37^ Occasionally, parenchymal abscess collections are seen as hypodense collections with mild rim enhancement, which are indistinguishable from non-tuberculous abscesses. These may also rupture into surrounding spaces, causing complications.^6,29^ Enhancing single or multiple renal parenchymal masses that do not communicate with the renal collecting system may be seen in ‘psuedotumour type’ (Figures 4 and 5).^6,29,36^

**FIGURE 6:**
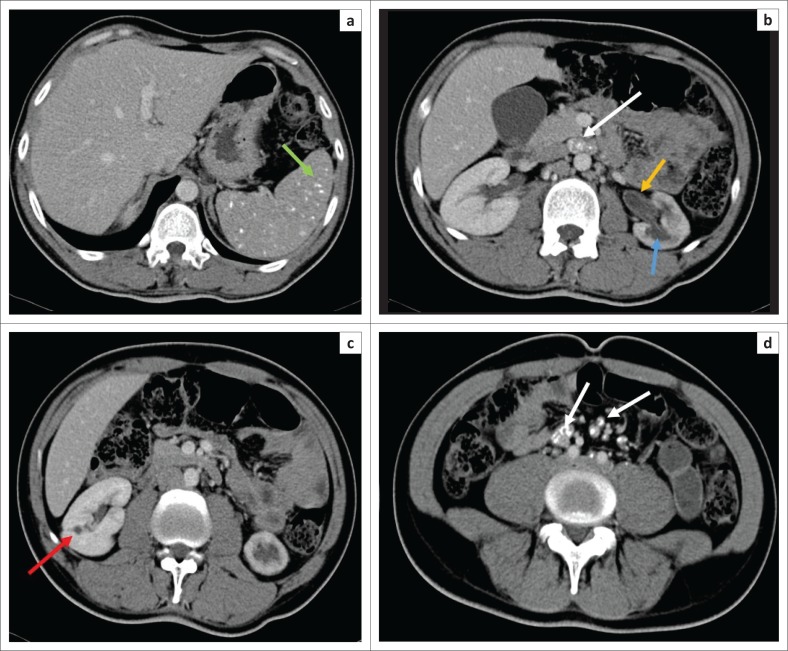
Post-contrast scan (venous phase) computed tomography (CT) abdomen demonstrates (a) multiple scattered calcified splenic granulomas (green arrow) and (b and d) calcified mesenteric and retroperitoneal lymph nodes (white arrows). (c) Small fluid-filled cavity in the right renal upper pole with adjacent cortical scarring (red arrow). The left kidney is shrunken with non-uniform calicosis (blue arrow) and adjacent parenchymal thinning. There is also prominence of the renal pelvis and thickening and enhancement of the urothelium (yellow arrow) suggestive of a pyelonephritis.

In the renal collecting system, early disease is demonstrated as irregularity of the renal calyces because of ulceration. Infundibular stenosis and amputation of the infundibulum with uneven hydrocalicosis are visualised on the excretory phase (Figures 4 and 6). Overlying parenchymal thinning and areas of parenchymal scaring may be noted (Figures 5 and 6).^2,3,6,29,33,36,37^ Focal or diffuse calicosis may be seen without dilatation of the renal pelvis.^29^ Urothelial inflammation demonstrates urothelial wall thickening and enhancement with surrounding fat stranding (Figures 5 and 6). A sawtooth appearance of the ureter may be seen because of ulceration. Multiple fibrotic strictures can present with a corkscrew appearance of the ureter. Coalescence of ureteric strictures results in straitening and a fixed appearance of the ureter, the ‘pipestem ureter’. Associated hydronephrosis and hydroureter are also evident.^6,29,33,36,37^ Bladder involvement causes mural thickening and enhancement because of cystitis. Bladder wall granulomas are noted as filling defects extending from bladder wall on the excretory phase.^6,33,36,37^

**FIGURE 7:**
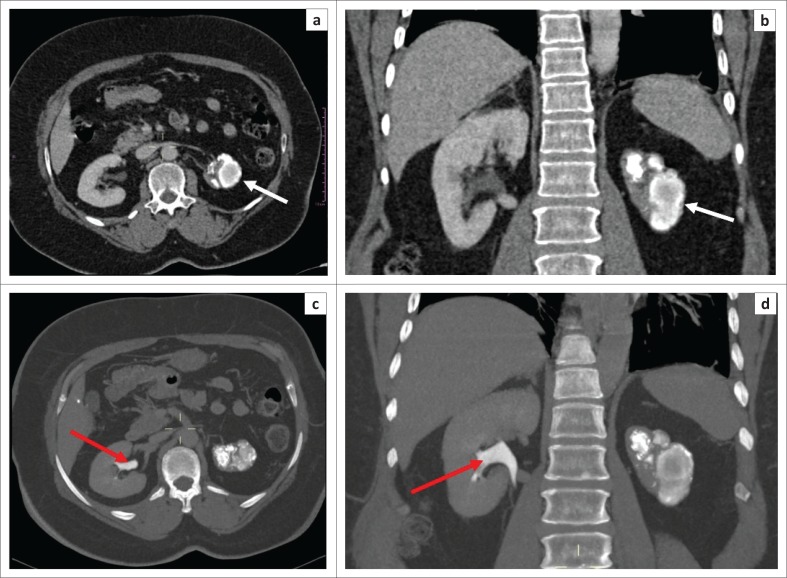
(a and b) Venous phase post-contrast computed tomography (CT) demonstrates a shrunken left kidney replaced by dystrophic calcifications (white arrow), in keeping with a ‘putty’ kidney. (c and d) Renal delay CT demonstrates contrast excretion in the right kidney (red arrow) and no contrast excretion in the non-functional left kidney, in keeping with autonephrectomy.

**FIGURE 8:**
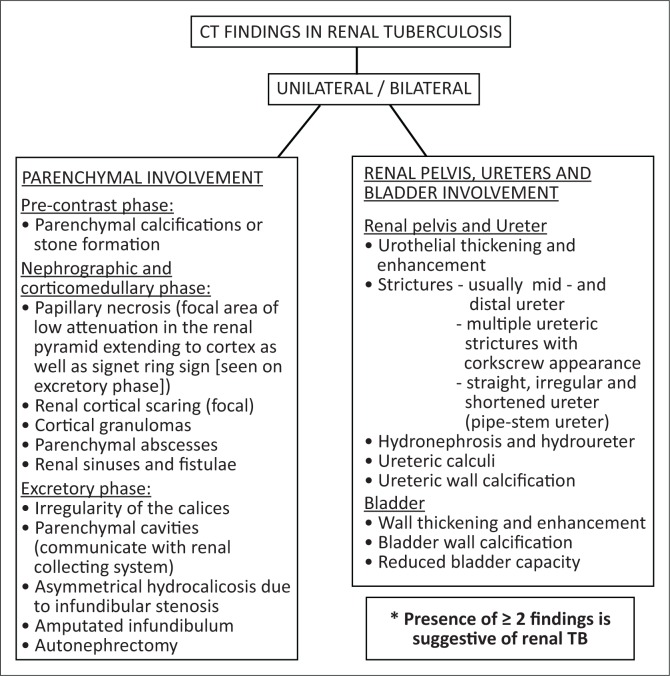
Computed tomography findings in renal tuberculosis.

The late MDCT findings include severe hydronephrosis with marked thinning of the renal cortex and loss of renal function because of chronic obstruction by pelvic or ureteric strictures (Figure 3).^3,6,29,36^ Destruction of the renal parenchyma with a shrunken kidney which is replaced by dystrophic calcifications is referred to as ‘putty kidney’ (Figure 7). This is end-stage disease and the kidney is non-functional at this stage with no excretion of contrast from the kidney, autonephrectomy.^6,24,36^ Amorphous, spotted, curvilinear, triangular or ring-like calcifications within the collecting system are characteristics of papillary necrosis. Lobar calcifications within the collecting system are in line with ‘putty kidney’.^6,24,29,33^ Chronic bladder involvement results in a shrunken bladder with low capacity, which is known as a thimble bladder in the severe form. Ureteral and bladder wall calcifications may be seen.^6,33,37^ Uncommon findings and complications include fistulae and sinus formation, which may appear as soft tissue tracts that may be filled with excreted iodine contrast with extensive perirenal inflammatory changes.^6,29^ Concomitant findings of extrapulmonary TB, such as hepatic and splenic granulomas, mesenteric and retroperitoneal lymph nodes, psoas abscess and spine findings may support the suspicion of renal TB (Figures 2 and 6).^2,3,29^ The MDCT findings of renal TB are further simplified in the chart provided in Figure 8.

The categories of reviewed articles included original research, case reports, literature review (radiological and non-radiological), organisational reports and grey literature. The original research reviewed pertaining to MDCT imaging of renal TB is summarised in Table 1. Moreover, one South African non-radiological original research article regarding renal TB was identified. In this article, Nourse et al. performed a study to assess the role of TB in children co-infected with HIV and concluded that TB contributed to proteinuric renal disease. However, it is not clear if this was because of direct infection of the kidney with Mycobacterium TB.

**TABLE 1:** Summary of original articles on multidetector computed tomography imaging of renal tuberculosis.

Author (Year of publication)	Study type	Country	Objectives	Sample size and demographics	Findings
Kulchavenya E. et al. (2013)	Retrospective	Novosibirsk - Siberia	To analyse age, gender and clinical spectrum of UGTB to improve its diagnostics.	*n* = 131 Male 81 (61.8%) Female 50 (38.2%)	Overall 75% KTB in UGTB spectrum 67.2% had isolated KTB Classification of kidney TB: Level 1 – non-destructive form, TB parenchyma Level 2 – small destructive form, TB papillitis Level 3 – destructive form with one or more caverns (cavernous TB) Level 4 – widespread destructive form with more than 2 caverns TB (polycavernous TB) >50% level 3 or 4 (older patients) Level 1 or 2 (younger patients)
Figueiredo A. et al. (2010)	Retrospective	Brazil	To assess the radiographic findings of UGTB of patients at different disease stages, for a better understanding of its pathophysiology.	*n* = 20 males (confirmed UGTB) Age 28–65 years (mean 41 years)	Bilateral renal TB with predominant parenchymal involvement = 1 AIDS patient Unilateral renal TB = 6 patients Unilateral renal TB with bladder TB = 6 patients Bilateral renal TB with bladder TB = 7 patients Most patients had associated ESRD at time of diagnosis Some had disease progression on follow-up despite stent placement and medical therapy
Satta S. et al. (2014)	Retrospective	Tunisia	To compare the presence and the frequency of imaging findings on IVP and CT To generate a systematic approach to imaging analysis of urinary TB	Total *n* = 159 (diagnosed with urinary TB) 46 analysed because both CT and IVP were performed Age 18–62 years 30 females; 16 males	Unilateral urinary TB – 42 (91%) IVP and 39 (85%) CT CT findings: - Hydronephrosis – 34 - Autonephrectomy – 27 - Ureteral thick wall – 18 - Hydroureter because of stricture – 14 Renal calcifications (CT) - Renal parenchyma – 4 - Urinary collecting system – 16 - Urinary collecting system wall – 3 Additional findings: - Psoas calcifications – 1 - Bone iliac TB – 1 - Psoas abscess – 5
Leung T.-K. et al. (2003)	Retrospective	Taiwan	To classify different imaging findings and clinical outcome in the ethnic communities represented by the cases.	*n* = 22	Renal TB was classified Mild = 13 - Early stage radiologic features - Normal renal function - Stable imaging findings at least 6 months after presentation Severe = 9 - Abnormal renal function not controlled by oral treatment - Surgery intervention - Irreversible renal damage
Wang L.-J. et al. (2003)	Retrospective	Taiwan	To analyse findings of IVP and CT in patients with urinary TB.	*n* = 53	No significant difference in depiction of moth-eaten calices, amputated infundibulum, autonephrectomy, urinary tract calcifications, renal parenchymal cavities and hydrocalicosis, hydronephrosis and hydroureter on IVU and MDCT
				47 – IVP; 33 – CT	Multiple findings in their patients were present in 94% of IVU and 100% of CT examinations
Guadiano C. et al. (2017)	Retrospective	Italy	To provide a guide for radiologist for searching the classic signs of UGTB on MDCTU, encouraging use of MPR and MIP.	*n* = 17	MDCT findings are included in Figure 8
				(confirmed UGTB)	Furthermore, they advocate use of MPR including curved MPR as well as use of MIP images and using bone settings when viewing MIP images to enhance early changes in the urothelium and calices
				Age 24–86 years (Mean 62)	

TB, tuberculosis; UGTB, urogenital tuberculosis; KTB, kidney tuberculosis; AIDS, acquired immunodeficiency syndrome; ESRD, end-stage renal disease; IVP, intravenous pyelogram; CT, computed tomography; MDCTU, multidetector computed tomography urography; MPR, multiplanar reconstruction; MIP, maximum intensity projection.

**FIGURE 9:**
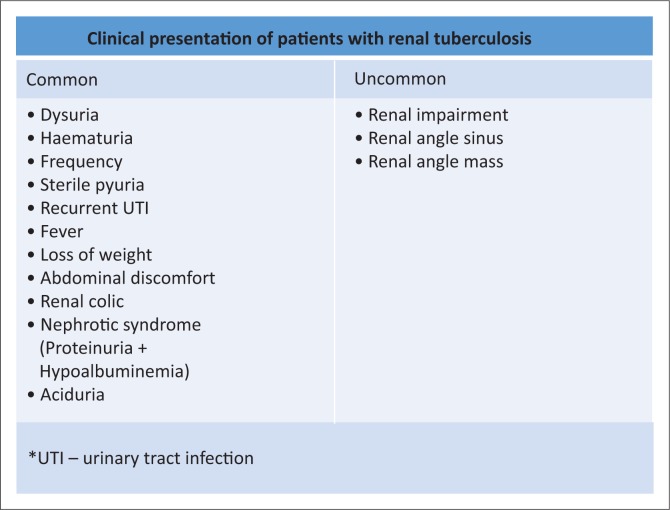
Clinical presentation of renal tuberculosis.

The clinical presentation of renal TB is summarised in Figure 9. Of the reviewed case reports, the common presentation was ESRD.^15,20,21^ Some of the patients were misdiagnosed, with the final diagnosis of renal TB being made months after initial presentation.^16^ Other presentations included a renal mass which was misdiagnosed as renal cell carcinoma. Rare presentations included a sinus in the back at the renal angle and paraspinal regions.^9,12,17,23,27^ A rare case of association of renal TB with renal tumour was identified.^38^ Few case reports of renal TB in paediatric patients were identified, including that of a 9-month-old.^9,18,23^ Complications of renal TB include formation of sinuses and fistulae, psoas abscess, stricture formation in the renal collecting system, renal failure as well as arterial hypertension.^23^ The patients discussed in the identified case reports did not have co-infection with HIV.

Kulchavenya^31,39^ has classified renal TB into the following four stages:

- Stage 1 – Non-destructive form (disease confined to the renal parenchyma. Full recovery of the kidney is possible).
- Stage 2 – Small destructive form (TB papillitis involving one or both sides; single or multiple. Responds to drug therapy. Prognosis is favourable).
- Stage 3 – Destructive form with one or two caverns (cavernous kidney TB involving one or both kidneys. It may require surgery).
- Stage 4 – Widespread destruction form with more than 2 caverns (polycavernous kidney TB which may lead to decreased renal function. Pyonephrosis may develop with formation of fistula. It may be self-limiting; the end result is autonephrectomy. Contralateral kidney is almost always involved. It almost always requires surgical treatment.).

He has also classified bladder TB as follows:

- Stage 1 – Infiltrative form (tubercles)
- Stage 2 – Ulcerous form (erosive)
- Stage 3 – Spastic cystitis (overactive bladder)
- Stage 4 – True microcystitis (shrunken bladder).

Although IVU is known to be more accurate in the evaluation of early changes in the renal collecting system, that is, moth-eaten calices, infundibular stenosis, amputation of the infundibulum and mucosal ulceration in the ureter, Gaudiano et al. demonstrated that the use of MPR and MIP images on excretory phase imaging can reproduce images comparable with IVU with high diagnostic accuracy, which is supported by Merchant et al. Interestingly, Wang et al. demonstrated no significant difference in the depiction of moth-eaten calices, amputated infundibulum, autonephrectomy, urinary tract calcifications, renal parenchymal cavities, hydrocalicosis, hydronephrosis and hydroureter on IVU and MDCT.

The imaging findings of renal TB are non-specific and have a long list of differential diagnosis; however, demonstration of multiple (≥ 2) findings is suggestive of the diagnosis.^2,7,10,21,29,33^ Wang et al. found the presence of multiple findings in their patients in 94% of IVU and 100% of CT examinations. Browne et al. recommend that any male patients presenting with UTI should be imaged using US as the initial imaging screening tool and women should be imaged after two to three episodes within a 12-month period. In addition, every male patient that is diagnosed with genital TB should be actively investigated for renal TB because of the strong association.^4^ They also advise that immunocompromised and diabetic patients should be imaged early.

The differential diagnosis for renal TB on MDCT includes other causes of chronic pyelonephritis and papillary necrosis, xanthogranulomatous pyelonephritis as well as renal cell carcinoma and transitional cell carcinoma.^10,12,15,16,21^ Schistosomiasis should be considered if there is bladder wall calcification; however, in contrast to urogenital TB, there is predominant involvement of the urinary bladder without destructive involvement of the upper urinary tract.^16,33,37^ Treatment of renal TB includes use of anti-TB therapy for a prolonged period, nephrectomy for non-functioning kidneys and renal masses and nephrostomy or stent placement for ureteric strictures to relieve obstruction.^2,9,11,12,16,19,27^ Fine needle aspiration (FNA) should be considered before performing nephrectomy in patients from an endemic area presenting with an atypical renal mass.^9,12,18,27,29^ This is an important consideration in young patients as renal tissue can be preserved if the diagnosis of TB is made preoperatively.^9^

A high radiation dose to the patient is the major concern in multiphase CT imaging of the urinary tract.^22,26,29,32,34^ The main aim of CT is to obtain images with opacification of the entire renal collecting system. However, this may be a challenge because of the presence of peristalsis resulting in non-opacification of some parts of the ureters, especially the mid and distal ureters.^32^ Current imaging protocols utilise three- or four-phase scans, which include an unenhanced scan and corticomedullary, nephrographic and excretory phase scan.^3,22,32,34^ Unenhanced images are used as baseline in the assessment of calcifications, Hounsfield unit (HU) of the lesions and degree of enhancement of renal lesions.^7,22,32^ Nephrographic phase is performed after intravenous injection of 100 mL–150 mL of 300 mg iodine contrast, at 100 s when both the cortex and medulla enhance. This phase allows adequate depiction of renal parenchymal lesions.^32^ The excretory phase is obtained to assess the urothelium. The nephrographic phase is omitted in the three-phase scan. Use of 250 mL of IV normal saline in adjunct with 10 mg of furosemide has been proven to improve distension and opacification of the renal collecting system; however, furosemide should not be used in patients with contraindications to the medication.^32^

**FIGURE 10:**
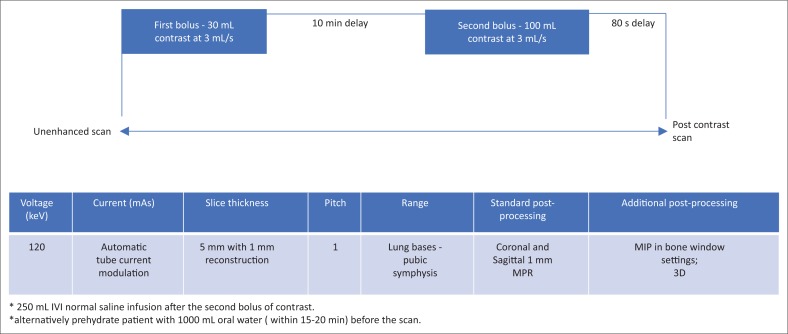
Recommended split-bolus computed tomography imaging protocol for diagnosis of renal tuberculosis.

Several options are available to reduce the radiation dose in CT renal imaging. One of them is by reducing the number of scans and performing only two phases, comprising an unenhanced scan and a split-bolus phase scan. In this method, initially, a small contrast bolus (30 mL) is administered IV followed by a larger bolus (100 mL–120 mL) after a 10–15 min delay. Computed tomography scanning is performed after the second contrast bolus (see Figure 10). This allows for a combination of excretory and corticomedullary phase in one scan. Other options include the use of an automatic tube current modulator, increasing pitch and slice thickness as well as reducing the scan field (i.e. covering the kidneys only for the corticomedullary phase).^34^

## Conclusion

Renal TB is one of the common manifestations of extrapulmonary TB. Diagnosis of the disease is challenging because of the non-specific presentation and a multidisciplinary approach will assist in early diagnosis. Radiologists can play a pivotal role in suggesting the diagnosis to the clinicians as early features are readily visualised on MDCT. Identification of acid-fast bacilli in the urine and histology specimens is however the gold standard for the diagnosis. Clinicians should consider including MDCT imaging when investigating patients with recurrent urinary tract infection, not responding to usual antimicrobial regimens. We suggest a two-phase MDCT imaging protocol which would increase the accuracy of the diagnosis while lowering radiation dose to the patients (Figure 10). We have simplified the MDCT findings of renal TB in the chart provided in Figure 8. We also emphasise the importance of using MPR and MIP images to enhance early pathology. Research in larger population groups, in endemic areas especially in Africa, is recommended to focus on assessing the accuracy of MDCT in early diagnosis of renal TB as well as the spectrum of MDCT manifestations in our population group, particularly those co-infected with HIV/AIDS. Studies in larger population groups are also recommended in endemic areas to assess the prevalence of renal TB in HIV-infected patients diagnosed with either pulmonary or disseminated TB.
